# Current career situations of Chinese pharmacovigilance professionals working for pharmaceutical companies: an exploratory survey

**DOI:** 10.1186/s12913-023-09089-0

**Published:** 2023-02-14

**Authors:** Yalan Tang, Yan Liu, Hongli Liao, Yonghua Yuan, Qihua Jiang

**Affiliations:** 1 The Department of Clinical Pharmacy, School of Pharmacy, Chongqing Medical University, Chongqing, China; 2 Chongqing Office, Beijing Captain Science Co., Ltd., Beijing, China; 3 The Department of Medical Chemistry, School of Pharmacy, Chongqing Medical University, Chongqing, China

**Keywords:** Pharmacovigilance professional, Career situation, Working condition, Mental pressure, Training, Competency, Turnover tendency

## Abstract

**Background:**

Pharmacovigilance in China has experienced rapid development in the past 30 years. The implementation of Good Pharmacovigilance Practice in China since the end of 2021 heralds a new era of pharmacovigilance affairs, which puts forward higher requirements for the quantity and quality of pharmacovigilance personnel. This study aimed to preliminarily explore the current career situations of pharmacovigilance professionals working in China for pharmaceutical companies.

**Methods:**

A questionnaire was adapted from research in the USA and Europe with the help of several pharmacovigilance experts. Snowball sampling was used to conduct an exploratory survey to obtain the frequency of basic demographic information, work status, and career expectations of pharmacovigilance professionals working for pharmaceutical companies.

**Results:**

The personnel engaged in pharmacovigilance work for pharmaceutical companies were mainly medical or pharmaceutical undergraduates within 3 years of graduation. Their work intensity and pressure were relatively high. The training provided by their universities and enterprises could not well meet their needs to improve their job competence. Although they were optimistic about pharmacovigilance and will not change their career, most of them were planning to change their employers.

**Conclusion:**

There was a gap between the demand and supply of pharmacovigilance personnel. Relevant regulatory authorities and industry associations should guide higher education institutions to collaborate with pharmacovigilance specialists to strengthen pharmacovigilance education for medical or pharmaceutical students, on the basis of which pharmacovigilance certification courses and continuing education courses can be developed. Meanwhile, pharmaceutical enterprises should consider reasonably adjusting work intensity and income to avoid a high turnover rate.

**Supplementary Information:**

The online version contains supplementary material available at 10.1186/s12913-023-09089-0.

## Background

World Health Organization (WHO) defined pharmacovigilance as “the science and activities relating to the detection, assessment, understanding, and prevention of adverse effects or any other medicine/vaccine related problems” [1]. So far the WHO Program for International Drug Monitoring (PIDM) has 152 full members and 22 associate members [2]. Since China officially joined the WHO-PIDM in 1998, China has made relatively great progress in legislation, organizational structure, information network system, and practice pertaining to pharmacovigilance. Following its first promulgation in 1999, the Measures for the Reporting and Monitoring of Adverse Drug Reaction (ADR) had two amendments in 2004 and 2011 respectively [3, 4]. Four-level (national, provincial, municipal, and county) ADR monitoring centers have been established [5–7], while hundreds of hospitals have been identified as sentinel sites of The National Adverse Drug Reaction Monitoring Sentinel Alliance Program [6, 7]. Two platforms, China Adverse Drug Reaction Monitoring System (CADRMS) and China Hospital Pharmacovigilance System (CHPS) have been supporting drug safety information collection and exchange. Annual ADR reports number rose from about 400 in 1999 via writing form to 1.96 million in 2021 via CADRMS [8]. The number of studies based on real-world Electronic Medical Records (EMRs) by use of CHPS has amounted to above 50 [9].

However, more than 80% of the ADR reports of 2021 were still submitted by healthcare professionals in hospitals [8], which was totally different from that of the USA, the UK, and Japan. The enforcement of Good Pharmacovigilance Practice by the National Medical Products Administration (NMPA) from December 1, 2021 lays down a set of requirements for marketing authorization holders (MAHs) and Drug Registration Applicants/Sponsors to conduct pharmacovigilance activities [10], which will make considerable demands for the quantity and specialty of pharmacovigilance professionals. There have been a series of researches chronicling the development of pharmacovigilance in China [5–7]. As for pharmacovigilance professionals, the focus of the researches was on: 1) the undergraduate or postgraduate education of pharmacovigilance [11–13], 2) the influence of new circumstances such as covid-19 and artificial intelligence [14, 15], 3) the attitude, knowledge, and behavior of ADRs report among medical staffs [16, 17]. Researchers rarely pay attention to the perception of the pharmacovigilance career by those choosing it and working for pharmaceutical companies.

The aim of this survey was to preliminarily explore the basic demographic features, working conditions, competency-based training, mental pressure, and views on the pharmacovigilance career of pharmacovigilance professionals working in China for pharmaceutical companies, the outcomes of which will be beneficial to pharmacovigilance human resources development and management.

## Methods

### Measurements

We designed an online survey to get information from pharmacovigilance professionals. The initial version of the questionnaire was adapted from research in the USA and Europe in 2017. Then it was sent to three pharmacovigilance experts for revision suggestions to ensure its applicability in China. After two rounds of revision, the final version of the questionnaire consists of 5 parts (Additional file 1). The first part contains 6 brief demographic queries about the gender, geographic location, age, educational level, specialty, and annual income of the respondents. The second section includes 6 questions focusing on the working conditions, such as employer types, working hours, business trips, and annual leave. The third part is based on a long-established mental pressure test scale which has been mainly used by the mental health center in West China Hospital of Sichuan University. The three-point Likert scale (never = 0, occasionally = 1, always = 2) with 17 items was used to evaluate the pressure of the pharmacovigilance workforce. The fourth part is comprised of 5 questions about the training and competencies of the professionals. The 6 questions in the last section aim to understand the perception of the present and future situations of pharmacovigilance by the respondents.

### Sample selection

The Survey link was sent to pharmacovigilance professionals via such social media as vocational WeChat Groups. It’s recommended to forward the link to the group members’ colleagues to execute a snowball sampling. All respondents were aware of the informed consent statement attached to the front part of the questionnaire and took part in the survey anonymously.

### Measuring technique

For each respondent, the scores of 17 items on the mental pressure test scale were added up to stand for his/her mental stress. The total pressure scores of each respondent were categorized into three kinds: low pressure (0–10), mild pressure (11–15), and heavy pressure (≥ 16).

### Data analysis

Data collected from April 2020 to May 2020 was exported from the questionnaire platform named questionnaire star and imported to Excel 2019 to be analysed. Incomplete questionnaires were excluded. Frequency analysis was conducted to describe the respondents’ demographic information, working conditions, training experience, and career prospects. The relation between pressure scores and annual income were illustrated by the percentages of three pressure types in different income groups.

## Result

### Demographic information

A total of 139 questionnaires were collected, of which 127 are valid and 12 are filled incomplete. The effective recovery rate of questionnaires was 91.37%. Table 1 demonstrates the demographic characteristics of the sample. Nearly 60% of the pharmacovigilance professionals were female. About 90% of the respondents worked in province-level municipalities or provincial capitals, among which 28.3% in the most prosperous 4 metropolises (Beijing; Shanghai; Guangzhou; Shenzhen). The largest proportion of pharmacovigilance professionals was those aged 20–29 years (74%). The percentages of respondents with Master’s and Bachelor’s degrees were 21.3% and 66.9% respectively. Pharmacy, clinical medicine, and nursing together accounted for almost 70% of the educational background. Another 23.6% of the respondents’ specialty was medicine or pharmacy related, for example, biology or chemistry. The top 3 annual income ranges were successively ¥50,000-¥100,000, ¥110,000-¥150,000, and ¥160,000-¥200,000.

**Table 1:** Demographic information of PV professionals sample in China in 2020 (*n* = 127)

Characteristic	No. of case	%
Gender		
Male	51	40.1%
Female	76	59.8%
Location		
Beijing; Shanghai; Guangzhou; Shenzhen	36	28.3%
Other province-level municipalities or provincial capitals	75	59.1%
Else cities	16	12.6%
Age		
20–29	94	74.0%
30–39	29	22.8%
40–49	4	3.1%
> 50	0	0.0%
Degree		
Doctorate	9	7.1%
Master’s	27	21.3%
Bachelor’s	85	66.9%
Diploma	6	4.7%
Specialty		
clinical medicine	20	15.7%
pharmacy	56	44.1%
nursing	15	11.8%
medicine/pharmacy related specialties	30	23.6%
Others	6	4.7%
Annual income		
< ¥50,000	16	12.6%
¥50,000-¥100,000	52	40.9%
¥110,000-¥150,000	32	25.2%
¥160,000-¥200,000	19	15.0%
¥210,000-¥300,000	8	6.3%
> ¥300,000	0	0.0%

### Working conditions

The Working conditions of the sample are displayed in Table 2. The proportion of respondents who had worked on pharmacovigilance for less than 1 year and 1–3 years were the same (35.4%), which was higher than that of those who had worked for 4–5 years (23.4%). 45.7% of the respondents worked for domestic pharmaceuticals, while 20.5% worked for foreign-owned pharmaceuticals. Another 15.7% were employed by outsourcing pharmacovigilance companies. The majority (90.6%) of respondents slept less than 7 h every day. Approximately 70% of the respondents worked more than 40 h every week. There was no business trip required for a little more than half (55.9%) of the respondents. A small proportion (5.5%) of the respondents needed to on business trips for above 6 days per month. Nonetheless, only 26.0% of the respondents could enjoy complete annual leave.

**Table 2:** Working conditions of PV professionals sample in China in 2020 (*n* = 127)

Characteristic	No. of case	%
Years work on pharmacovigilance		
< 1	45	35.4%
1–3	45	35.4%
4–5	30	23.6%
6–10	7	5.5%
> 10	0	0.0%
Employer type		
foreign-owned pharmaceutical company	26	20.5%
domestic pharmaceutical company	58	45.7%
outsourcing pharmacovigilance company	20	15.7%
self-employment	23	18.1%
Sleep hours every day		
< 4 h	7	5.5%
4-5 h	44	34.6%
6-7 h	64	50.4%
> 7 h	12	9.4%
Working hours per week		
< 40 h	40	31.5%
40-60 h	79	62.2%
> 60 h	8	6.3%
Days on business trip per month		
> 15d	0	0.0%
11-15d	2	1.6%
6-10d	5	3.9%
1-5d	49	38.1%
Never	71	55.9%
Annual leave		
never	10	7.9%
seldom	26	20.5%
sometimes, shorter than normal	58	45.7%
always, as long as normal	33	26.0%

### Mental pressure

**Fig. 1:**
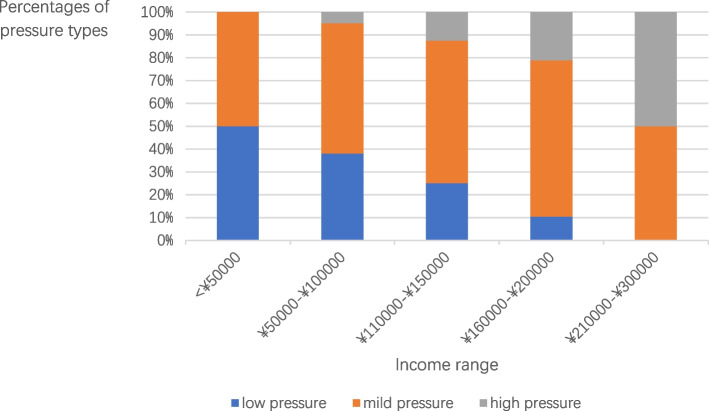
Percentages of three pressure types in different income ranges of PV professionals sample in China in 2020 (*n* = 127)

As shown in Table 3, 62.2% of the respondents were under mild pressure and 11% felt high pressure. Merely 26.8% faced low pressure. The relation between pressure scores and income was obvious, while other relations between pressure scores and other variables including age and sex are subtle. Figure 1 illustrates the mental pressure of different income groups. It’s obvious that the percentage of high pressure rose with the increase in income, whereas that of low pressure went the opposite.

**Table 3:** Scores of pressure test of PV professionals sample in China in 2020 (*n* = 127)

Score	No. of case	%
0–10(low pressure)	34	26.8%
11–15(mild pressure)	79	62.2%
≥ 16(high pressure)	14	11.0%

### Training and competencies

Table 4 displays the current status of training and competencies of respondents. About 3 quarters (76.4%) of them had gotten some instruction on adverse drug reactions or pharmacovigilance in their university. 40.9% of the respondents’ companies laid ordinary emphasis on pharmacovigilance affairs, roughly equal to the sum of which laid great and strong emphasis. The percentages of the respondents claiming their employer supplied frequent and occasional training in pharmacovigilance skills and competencies were 33.1% and 63.8% respectively. Despite this, nearly 90% of the respondents asserted a demand for enhancing professional competencies. Above 90% of the respondents reckoned professional competencies include knowledge of pharmacovigilance legislation and regulatory requirements, MedDRA and WHODRUG, clinical therapy, and safety data utilization. There were comparable respondents who underlined the proficiency in technical safety systems. Meanwhile, most respondents considered good communication ability essential.

**Table 4:** Training and competencies of PV professionals sample in China in 2020 (*n* = 127)

	No. of case	%
Have you ever been trained in adverse drug reactions or pharmacovigilance in your university?		
yes	97	76.4%
no	30	23.6%
How much emphasis does your company place on pharmacovigilance affairs?		
strong	17	13.4%
great	46	36.2%
ordinary	52	40.9%
a little	8	6.3%
little	4	3.2%
How often does your company train pharmacovigilance professionals for skills and competencies?		
frequently	42	33.1
occasionally	81	63.8
rarely	4	3.1
How do you need to enhance professional competencies?		
need much	31	24.4%
need some	84	66.1%
not need much	10	7.9%
no need	2	1.6%
What competencies do you think you have to help you perform your tasks in your role?		
Knowledge of applicable national Pharmacovigilance legislation (GVP, ICH-GCP)	116	91.3%
Understanding of global regulatory requirements for Pharmacovigilance (FDA, EMA, TGA)	116	91.3%
Proficiency in technical safety systems	116	91.3%
Working knowledge of MedDRA and WHODRUG coding dictionaries and medical terminology	116	91.3%
Clinical knowledge of therapeutic area patient populations	116	91.3%
Knowledge of aggregate safety data utilization	116	91.3%
Good communication ability	114	89.8%

### Opinions on pharmacovigilance career

The respondents’ opinions on the existing state and prospects of pharmacovigilance career are in Table 5. Over 90% of the surveyees felt the special attention was paid to pharmacovigilance by the government and society. Over 95% of respondents confirmed vacancies for pharmacovigilance in their companies and were satisfied with their working environment. Nevertheless, 83.5% of the respondents had ever thought about resigning from their companies, and 77.2% of them expressed an intention to change their career. Surprisingly, nearly 95% of the respondents see a positive future for pharmacovigilance development.

**Table 5:** Opinions of PV professionals sample in China in 2020 on the present state and prospects of pharmacovigilance (*n* = 127)

	No. of case	%
How often do you feel the emphasis placed on pharmacovigilance by the country and society?		
frequently	27	21.3%
occasionally	90	70.8%
rarely	10	7.9%
How about the vacancies for pharmacovigilance in your company?		
maximum	85	66.9%
minimum	36	28.4%
none	6	4.7%
How are you satisfied with your work environment?		
totally satisfied	16	12.6%
very satisfied	33	26.0%
satisfied	72	56.7%
dissatisfied	5	3.9%
totally dissatisfied	1	0.8%
Have you ever thought about leaving your current company?		
usually	17	13.4%
sometimes	89	70.1%
never	21	16.5%
Have you ever thought about changing your current career?		
usually	7	5.5%
sometimes	91	71.7%
never	29	22.8%
How do you expect the pharmacovigilance career in the future?		
prosperous	24	18.9%
promising	28	22.1%
good	68	53.5%
not ideal	7	5.5%
bad	0	0.0%

## Discussion

### Domestic pharmaceutical companies

Among the respondents, the ratio of the number of people working for foreign-owned pharmaceutical companies to that of people working for domestic ones is approximately 1:2, which is much higher than the ratio of these two kinds of pharmaceutical companies in China [18]. One reason is that China allows pharmacovigilance work to be outsourced, considering the actual situation of China's pharmaceutical industry [10]. Some domestic companies have outsourced pharmacovigilance work to reduce the number of related staff needed. Another reason for this phenomenon is that foreign enterprises need more pharmacovigilance professionals due to the large number of their innovative drugs. However, with the increasing innovation level of China's pharmaceutical industry, domestic enterprises need to have due regards to pharmacovigilance work, by either establishing their own team of pharmacovigilance personnel or accumulating experience in the selection of qualified outsourcing pharmacovigilance companies.

### Pharmacovigilance training and education

The majority of respondents were young. Most of them had bachelor's degrees in medicine or pharmacy and had worked in pharmacovigilance for less than 3 years. Some of the respondents received barely any knowledge of adverse drug reactions or pharmacovigilance in their university education. Despite widespread in-house training by pharmaceutical companies, respondents still felt the need to upgrade their knowledge and skills in all aspects of pharmacovigilance. Therefore, it can be inferred that there exists a huge demand for education and training in pharmacovigilance.

In fact, there were flourishing pharmacovigilance training courses, conferences, and seminars in China in recent years. The International Society of Pharmacovigilance (ISoP) and The Uppsala Monitoring Center of WHO (WHO-UMC) held pharmacovigilance joint courses with Shenyang Pharmaceutical University, Shenzhen Hospital of Hong Kong University, and NMPA in January 2018, November 2019, and October 2021, respectively [19], after the Chinese drug regulatory authority’s membership in the International Council for Harmonisation of Technical Requirements for Pharmaceuticals for Human Use (ICH). Nevertheless, the direct impact of these courses was limited in scope and depth because these courses lasted only 1–2 days each time.

Chinese colleges and universities haven’t extensively taken part in pharmacovigilance course development. To our knowledge, Shenzhen University and Shenzhen Pharmacovigilance Institute jointly offer an individual Pharmacovigilance and Risk Management course [20]. Besides, Jiaxing University collaborated with Jiaxing Taimei Medical Technology Co., LTD. to offer an independent Pharmacovigilance course to their medical students [21].

Priority should be given to integrating professional seminars and university courses into the internal pharmacovigilance training of pharmaceutical companies. It’s feasible for the relevant regulatory authorities and industry associations to encourage colleges and universities to vigorously develop pharmacovigilance education. In addition to distinguished pharmacovigilance monographs, what can be used for reference includes the WHO-UMC online courses [22], the European training programme in pharmacovigilance and pharmacoepidemiology (EU2P) [23], and the ISoP Global Pharmacovigilance Professional Certification (GPPC) project [24]. To ensure the courses are up to date, experienced pharmacovigilance experts should be attracted to participate in the construction of localized pharmacovigilance education. The initial courses may focus on medical or pharmacy undergraduates, which can be upgraded and transformed into vocational certification courses and continuing education courses, particularly if online learning platforms are used to facilitate the study.

### Work intensity and turnover tendency

It can be seen from the sleep time, business trip, and annual leave of the respondents that pharmacovigilance professionals had somewhat great work intensity and pressure increases with income. Most of the respondents were willing to leave their company, even though they were satisfied with the working environment and optimistic about the prospect of the pharmacovigilance career thus less willing to change their career. One of the reasons for the high work intensity and pressure is the lack of pharmacovigilance personnel at present. The large number of job vacancies for pharmacovigilance in enterprises prompts the on-the-job personnel to bear more work. In addition to the work intensity and pressure, the strong turnover intention may be caused by the fact that the annual salary of most respondents working in highly developed cities is lower than the local average salary. It’s in turn possible to obtain more income by changing employers in the same industry because of the high job vacancies.

A reasonable mobility of pharmacovigilance personnel is helpful to the development of the industry, but frequent job-hopping requires a long adaptation period, resulting in the waste of human resources. Therefore, the education and training of new pharmacovigilance personnel to fill job vacancies, hence reducing labor intensity and pressure has fundamental help. Under the background of the declining employment rate of medical students in China [25], it is suggested that more medical graduates be attracted to work on pharmacovigilance in the future. In addition, pharmaceutical companies should also pay attention to workforce protection such as working hours and wage optimization.

### Limitations

The sampling was conducted before the implementation of Good Pharmacovigilance Practice which will undoubtedly have quite an impact on pharmacovigilance practice. However, the survey results can be a reflection of current and even near-future conditions because adaptation by the pharmaceutical industry is difficult to be completed in a short period. Snowball sampling may affect the representativeness to a certain extent. Still, it is acceptable for acceleration access to the sample. Besides, this study is an exploratory study aiming to reveal the overall situation of pharmacovigilance professionals. It merely provides some basis for further in-depth research, including but not limited to the investigation of pharmacovigilance training needs and the factors of pharmacovigilance staff work stress.

## Conclusion

This survey shows that pharmacovigilance professionals working for pharmaceutical enterprises in Chinese are mostly medical or pharmaceutical bachelors who have recently graduated, and their work intensity and pressure are relatively high. The implementation of Chinese Good Pharmacovigilance Practice will increase the shortage of qualified pharmacovigilance personnel. In this circumstance, colleges and universities should speed up the construction of pharmacovigilance training courses, including undergraduate, vocational certification, and continuing education courses, together with experienced experts in pharmacovigilance practice. Domestic pharmaceutical enterprises, especially innovative drug companies, should pay attention to the construction of their own pharmacovigilance system. At the same time, enterprises should take into consideration reasonable adjustments of labor intensity and income to avoid the influence of a high turnover rate on the continuity of pharmacovigilance work.

## Supplementary Information

**Additional file 1**. Questionnaire.

## Data Availability

All data generated or analysed during this study are included in this published article.

## Abbreviations

WHO: World Health Organization
PIDM: Program for International Drug Monitoring
ADR: Adverse Drug Reaction
CADRMS: China Adverse Drug Reaction Monitoring System
CHPS: China Hospital Pharmacovigilance System
EMRs: Electronic Medical Records
NMPA: National Medical Products Administration
MAHs: Marketing authorization holders
ISoP: The International Society of Pharmacovigilance
UMC: Uppsala Monitoring Center
ICH: International Council for Harmonisation of Technical Requirements for Pharmaceuticals for Human Use
EU2P: European training programme in pharmacovigilance and pharmacoepidemiology
GPPC: Global Pharmacovigilance Professional Certification project
