# Supplementary material for: Current career situations of Chinese pharmacovigilance professionals working for pharmaceutical companies: an exploratory survey
Source: BMC Health Serv Res. 2023 Feb 14;23:152. doi: 10.1186/s12913-023-09089-0 (PMC9926404; doi:10.1186/s12913-023-09089-0)
Supplement: Supplementary file 1 — Additional file 1. Questionnaire. [file 12913_2023_9089_MOESM1_ESM.docx]

**QUESTIONNAIRE**

Current career situations of Chinese pharmacovigilance professionals working for pharmaceutical companies

**Section 1**

**Q1:**

What’s your gender？

A. Male B. Female

**Q2:**

What’s your location？

A. Beijing; Shanghai; Guangzhou; Shenzhen

B. other province-level municipalities or provincial capitals

C. else cities

**Q3:**

What’s your age？

A. 20-29 B. 30-39 C. 40-49 D. >50

**Q4:**

What’s your final university degree?

A. Doctorate B. Master’s C. Bachelor’s D. Diploma

**Q5:**

What’s your specialty?

A. clinical medicine B. pharmacy C. nursing D. medicine/pharmacy related specialties

E. Others

**Q6:**

What’s your annual income?

A. <¥50000 B. ¥50000-¥100000 C. ¥110000-¥150000 D. ¥160000-¥200000

E. ¥210000-¥300000 F. >¥300000

**Section 2**

**Q7:**

How many years of pharmacovigilance work experience do you have?

A. <1 B. 1-3 C. 4-5 D. 6-10 E. >10

**Q8:**

What’s the type of your employer?

A. foreign-owned pharmaceutical B. domestic pharmaceutical

C. outsourcing pharmacovigilance company D. self-employment

**Q9:**

How long do you sleep every day?

A. <4h B. 4-5h C. 6-7h D. >7h

**Q10:**

How many hours do you work every week?

A. <40h B. 40-60h C. >60h

**Q11:**

How long is the average monthly business trip?

A. >15d C. 11-15d D. 6-10d E. 1-5d F. never take business trips

**Q12:**

How do you use your annual leave?

A. never B. seldom C. sometimes, shorter than normal D. always, as long as normal

**Section 3**

**Q13:**

Stress test scale by the mental health center in West China Hospital of Sichuan University

Please recall the following situation in the past month

Q13-1 feel there is too much work to accomplish

A. never B. occasionally C. always

Q13-2 feel the time is not enough and every minute counts, eg. run a red light when crossing the road, walk and talk quickly

A. never B. occasionally C. always

Q13-3 think about work all day long, thus feel no time for entertainment

A. never B. occasionally C. always

Q13-4 easy to lose temper when you are frustrated

A. never B. occasionally C. always

Q13-5 worry about other’s evaluation of your work performance

A. never B. occasionally C. always

Q13-6 don’t think the superiors and family members appreciate me

A. never B. occasionally C. always

Q13-7 worry about my economic status

A. never B. occasionally C. always

Q13-8 need to treat headache, back pain, or stomach pain

A. never B. occasionally C. always

Q13-9 need to use alcohol, drugs, food, etc. to suppress restlessness

A. never B. occasionally C. always

Q13-10 need sleeping pills to fall asleep

A. never B. occasionally C. always

Q13-11 lose temper when getting along with family, friends, and colleagues

A. never B. occasionally C. always

Q13-12 interrupt others when they are confiding

A. never B. occasionally C. always

Q13-13 feel so upset about the unfinished business that I can’t fall asleep.

A. never B. occasionally C. always

Q13-14 have too much work to make everything perfect.

A. never B. occasionally C. always

Q13-15 feel guilty about relaxation even in the free time.

A. never B. occasionally C. always

Q13-16 impatient and feel guilty after acting willfully

A. never B. occasionally C. always

Q13-17 think myself shouldn’t have fun

A. never B. occasionally C. always

**Section 4**

**Q14:**

Have you ever been trained in adverse drug reactions or pharmacovigilance in your university?

A. yes B. no

**Q15:**

How much emphasis does your company place on pharmacovigilance affairs?

A. strong B. great C. ordinary D. a little E. little

**Q16:**

How often does your company train pharmacovigilance professionals for skills and competencies?

A. frequently B. occasionally C. never

**Q17:**

How do you need to enhance professional competencies?

A. need much B. need some C. not need much D. no need

**Q18:**

What competencies do you think you have to help you perform your tasks in your role?

Skills

A. Knowledge of applicable national Pharmacovigilance legislation (GVP, ICH-GCP)

B. Understanding of global regulatory requirements for Pharmacovigilance (FDA, EMA, TGA)

C. Proficiency in technical safety systems

D. Working knowledge of MedDRA and WHODRUG coding dictionaries and medical terminology

E. Clinical knowledge of therapeutic area patient populations

F. Knowledge of aggregate safety data utilization

G. Good communication ability

Section 5

**Q19**

How often do you feel the emphasis placed on pharmacovigilance by the country and society?

A. usually B. sometimes C. rarely

**Q20:**

How about the vacancies for pharmacovigilance in your company?

A. maximum B. minimum C. none

**Q21:**

How are you satisfied with your work environment?

A. totally satisfied B. very satisfied C. satisfied D. dissatisfied E. totally dissatisfied

**Q22:**

Have you ever thought about leaving your current company?

A. usually B. sometimes C. never

**Q23:**

Have you ever thought about changing your current career?

A. usually B. sometimes C. never

**Q24**

How do you expect the pharmacovigilance career in the future?

A. prosperous B. promising C. good D. not ideal E. bad
